# Proposal for a descriptive and differentiated presentation of the longitudinal impact of the new organized cancer screening guideline and HPV vaccination in Germany

**DOI:** 10.1007/s00404-022-06747-2

**Published:** 2022-09-02

**Authors:** F. Neis, B. Holleczek, M. Henes, I. Juhasz-Böss, D. Wallwiener, K. J. Neis

**Affiliations:** 1 Department of Obstetrics and Gynecology, University Hospital Tübingen, Calwerstrasse 7, 72076 Tübingen, Germany; 2 Saarland Cancer Registry, Saarbrücken, Germany; 3 Department of Obstetrics and Gynecology, University Hospital Freiburg, Freiburg, Germany; 4 Frauenärzte am Staden, Saarbrücken, Germany

**Keywords:** Cervical screening, Co-test, CIN 3, Longitudinal effects, Organized cancer screening guideline, oKFE-RL, HPV vaccination

## Abstract

**Introduction:**

Since 01/01/2020, the cervical cancer screening in Germany has been carried out due to the organized early cancer diagnosis guideline (oKFE-RL). In 2007, HPV vaccination was initiated in Germany. The main goal of both initiatives is to further reduce the incidence of invasive cervical cancer. To assess the effect of the new screening strategy in a timely manner, monitoring of short-term changes need to be considered. Ideally, the effects of both prevention methods would be presented together in one model.

**Materials and methods:**

Because no change in the incidence of invasive cervical cancer is initially expected, the incidence of CIN 3 is used as a surrogate parameter to assess the effects of the prevention efforts. Based on expected additional effects of vaccination and co-testing, a model-based estimation of the expected CIN 3 incidence during the evaluation of the screening program is performed using the CIN 3 incidence in the Saarland population.

**Modeling results:**

The oKFE-RL provides for two groups: Primary cytodiagnosis continues until 35 years of age. Here, in the next few years, CIN 3 incidence will be reduced not by the oKFE-RL but by the increasing proportion of vaccinated women. In the group over 35 years, co-testing was introduced with a stringent algorithm. Due to the higher sensitivity of the HPV test, significantly more CIN 3 are detected in the first round of 3 years and thus, the CIN 3 incidence initially increases. As these CIN 3 are absent in the second round, significantly fewer CIN 3 cases will be detected then. These effects suggest a global decrease in CIN 3 incidence of 25.8% after 6 years.

**Conclusion:**

Observation of the age distribution curve of CIN 3 allows both effects of prevention to be assessed in a timely manner and separately. In the future, data from epidemiologic cancer registries should be incorporated into the model to replace modeling with real data.

## What does this study add to the clinical work

**Table:** 

CIN 3 and invasive cervical cancer are the target lesions of HPV vaccination and the newly established co-test in Germany. In the presented model, both influences can be shown separately in the age distribution curve of CIN 3.	

## Introduction

On 01.01.2020, a new screening strategy for the early detection of cervical cancer was implemented in Germany [organized cancer screening guideline (oKFE-RL)] [1]. Thereby, the screening population is divided into two groups: women from 21 to 35 years of age continue to receive annual cytologic diagnosis due to the high prevalence of human papillomavirus (HPV) in this age group. Women 36 years of age and older are offered co-testing (cytology and HPV testing) at a 3-year interval. In the female population between the ages of 30 and 35, a transition group will be created in which HPV testing will be integrated into the algorithm for clarifying abnormal findings. At the same time, women vaccinated against HPV as adolescent are entering constantly into the first population group of women who exclusively got the cytological diagnostics.

Regarding the effects of the interventions implemented according to the oKFE-RL medical history, cytodiagnostics, HPV test, colposcopy, result of a biopsy as well as the surgical interventions indicated by these will undergo an evaluation according to the specifications of the German Institute for Quality Assurance and Transparency in Health Care (IQTIG), as specified by the Federal Joint Committee (G-BA). The aim is to record and illuminate the quality, effectiveness and efficiency of organized cancer screening [2].

Unlike other cancer screening programs (such as mammography screening), cervical cancer screening aims to reduce not only mortality but also incidence. This is because this particular screening has also primary preventive potential: by detecting and eliminating the direct precursor lesions of invasive cervical cancer, cervical intraepithelial neoplasia grade 3 (CIN 3) and adenocarcinoma in situ (AIS), the development of invasive cancer can be prevented to a large extent. Hereby, in the longer term, the incidence of malignant neoplasms of the cervix can be reduced [2].

The time period between the occurrence of CIN 3 and the resulting invasive cervical cancer is estimated to be 10–20 years or more, with approximately half of all untreated CIN 3 having progressed to cervical cancer by the end of 30 years [3]. Since, nowadays, life expectation of women is at about 80 years, it can be expected that even a higher proportion of cervical cancer based on an early infection will still develop later in life [4].

In light of this, it is clear that within the advised timeframe of the evaluation of cervical cancer screening of 6 years (2020–2026), no prediction about the reduction in the incidence of invasive cervical cancer can be made. Instead, the incidence of CIN 3 needs to be used as a surrogate parameter, as it has been used in numerous studies in the past [5–7].

The aim of this work is to develop a model to estimate the expected trends in the incidence of CIN 3 in the general population as a surrogate parameter for the effectiveness of the different prevention measures: HPV vaccination and screening by cytology and co-testing. Thus, a comprehensive picture of the impact of both prevention methods, which have the same goal of reducing the incidence of cervical cancer, can be created.

## Materials and methods

The Saarland Epidemiological Cancer Registry (ECR) records all invasive carcinomas occurring in the population, especially cervical cancer, and its precursor CIN 3, for about 50 years. The population of Saarland can be considered sufficiently representative for the population of Germany. Accordingly, over a long period of time, the data on the cancer burden of the population in Saarland were used as a basis for estimating cancer incidence and mortality for the Federal Republic of Germany [8]. The Saarland Cancer Registry is currently confirmed to have a registration completeness of nearly 100%.

**Fig. 1:**
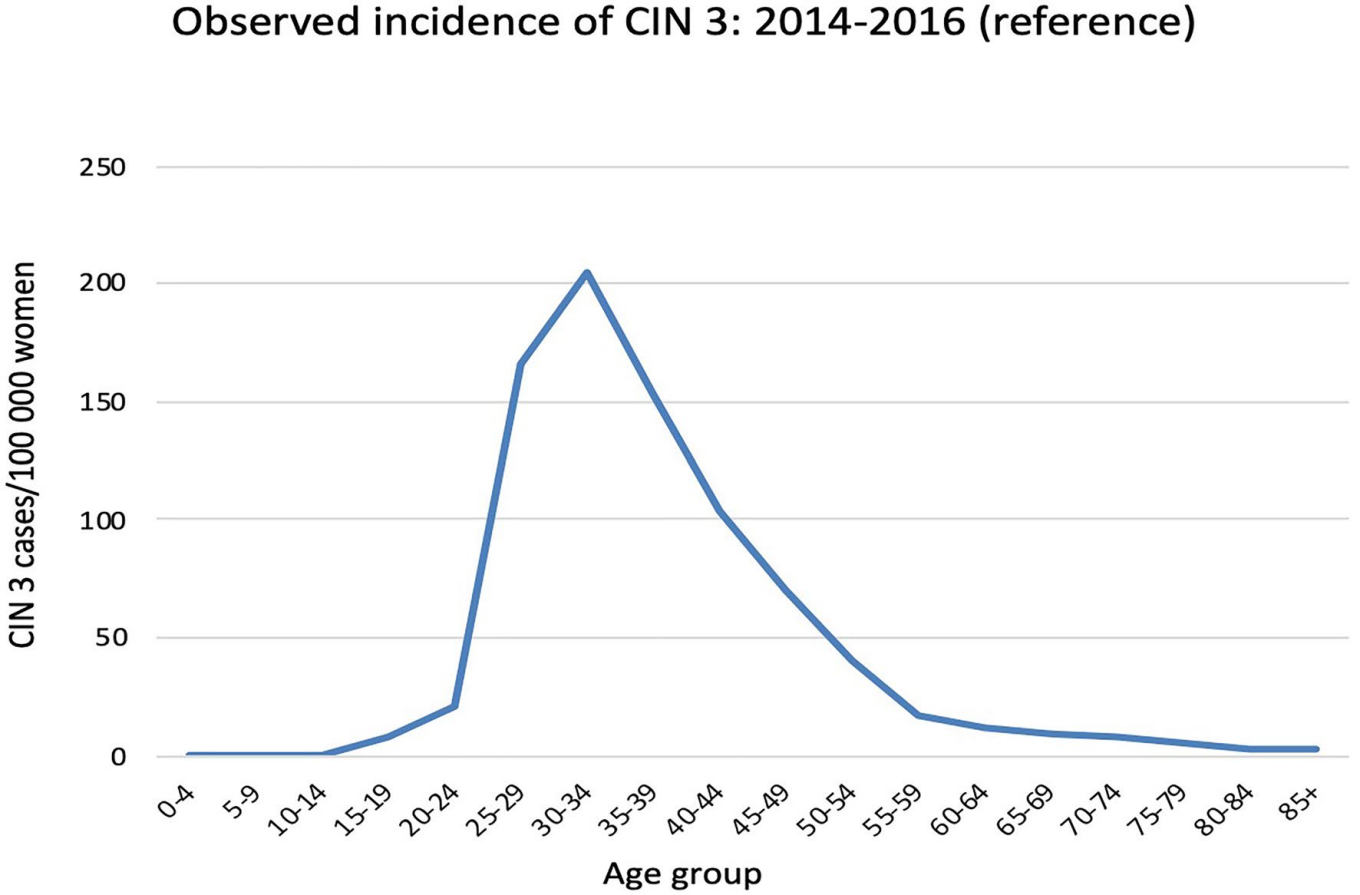
Age distribution of CIN 3 cases per 100 000 women in Saarland (Germany) 2014–2016 (baseline curve). Total number of CIN 3 in the time period 2014–2016: *N* = 773. Incidence across all age groups: 53.9/100,000 CIN 3 cases

The age-specific distribution of CIN 3 has undergone a continuous change over the last 50 years and shows a stable curve in the last 20 years. This correlates with the slowly decreasing incidence of invasive carcinoma during this time period [9]. In the few other countries where CIN 3 is also recorded, this is visible in the same way. For example, the age distribution curve from the United Kingdom in the period of 2015–2017, with a total of 85,000 CIN 3 cases [10] shows almost the same pattern as the Saarland curve from 2014 to 2016 with 733 CIN 3 cases (Fig. 1).

Against this background, the data on the age-specific incidence of CIN 3 or in situ carcinomas of the cervix (ICD-10 code: D06) determined in Saarland in the period 2014–2016 can be used as a reference (so-called baseline curve) for Germany. In the ICD-10 system, endocervical and ectocervical high-grade lesions of the cervix uteri are coded as D06. A discrimination between squamous and glandular lesions is not made. In the documentation of the Saarland ECR, all lesions are therefore documented as CIN 3. Since a further breakdown is not possible, this paper only refers to CIN 3, knowing that among these data patients with AIS are also included.

As CIN 3 data have been increasingly collected in all other German ECRs for several years, these data will be comprehensively available in the near future. Subsequently, it will be possible to incorporate these results into the baseline curve. To show the influence of the new oKFE-RL as well as the HPV vaccination on the incidence of CIN 3 and, thus, on the curve progression in our model, we have estimated the expected changes in advance. This first step is intended to illustrate the development in the first two rounds as an exemplary model:

Within this curve there can be built two groups, which will overlap not before 2030. The group of women between the 21st and 35th year of life with vaccination influence can be separated exactly from the group of the women older than 35 years, with whom a changing of the curve is expected due to the using of the co-testing. An another advantage of the model proposed by us is that these illustrations do not require complex epidemiological calculations, which in turn benefits the general understanding as desired by the national cancer plan [11].

### Vaccination

In 2007, the HPV vaccination started in Germany and all over the world. Then, the girl's age has been 12 years on the average. Thus, they are to be allocated the year of birth of 1995. In 2015, they stepped into the screening at the age of 20 years and they will have reached the 30th year of life in 2025.

For this population group (21st–35th years of life), we assume the following: according to data of the Robert-Koch Institute, the average 18-year-old girls (year of birth 2001) with complete vaccination in 2019 was at 52.0% [12]. In a Cochrane-Analysis, Arbyn described the efficiency factor of vaccination between 98 and 99% [13]. Drolet showed in her Systematic Review a reduction of the prevalence of HPV 16 and 18 of 66–83% [14].Other authors act on the assumption of a smaller effect [15–19]. We assumed a 95% effect in our model.

The HPV genotypes, primarily relevant for the development of CIN 3 are the types 16 and 18, which are included in all 3 available vaccines [15]. These genotypes occur in approximately 80–90% of CIN 3 in the age group of 21–34 years of age [5, 20, 21].

If all of the above factors are combined, based on the current situation in Germany, this results in a probable reduction of the incidence of CIN 3 by vaccination of about 35% within 3 years in the group of women with complete access to vaccination (Table 1). Within the 1st evaluation round, there are only 40% with complete access to the HPV vaccination in the group of the 25–29-year-old women (Table 2: green boxes, Round 1), so we estimated the effect at 14% in the first round. In the further period, the proportion of women with complete access to vaccination in this group increases to 93%, so the effect is more distinct here (Table 2: green boxes, Round 2). Therefore, we estimated the effect to be 33% in round 2 in the group of 25–29-year-old women.

**Table 1:** Group-specific estimated changes in the incidence of CIN 3 in rounds 1 and 2 in contrast to the baseline curve

Round	Age group (years)	Estimated change (%)	Rationale
Round 1	< 25	− 35	Vaccination effect
	25–29	− 14	Only 40% with access to HPV vaccination, therefore only proportional reduction
	30–34	+ 15	Cytology + reflex HPV testing
	> 35	+ 30	Co-test
Round 2	< 25	− 35	Vaccination effect
	25–29	− 33	only 93% with access to HPV vaccination, therefore only proportional reduction
	30–34	− 18	Cytology + reflex HPV testing, and first vaccinated cohort
	> 35	− 30	Co-test

**Table 2:**
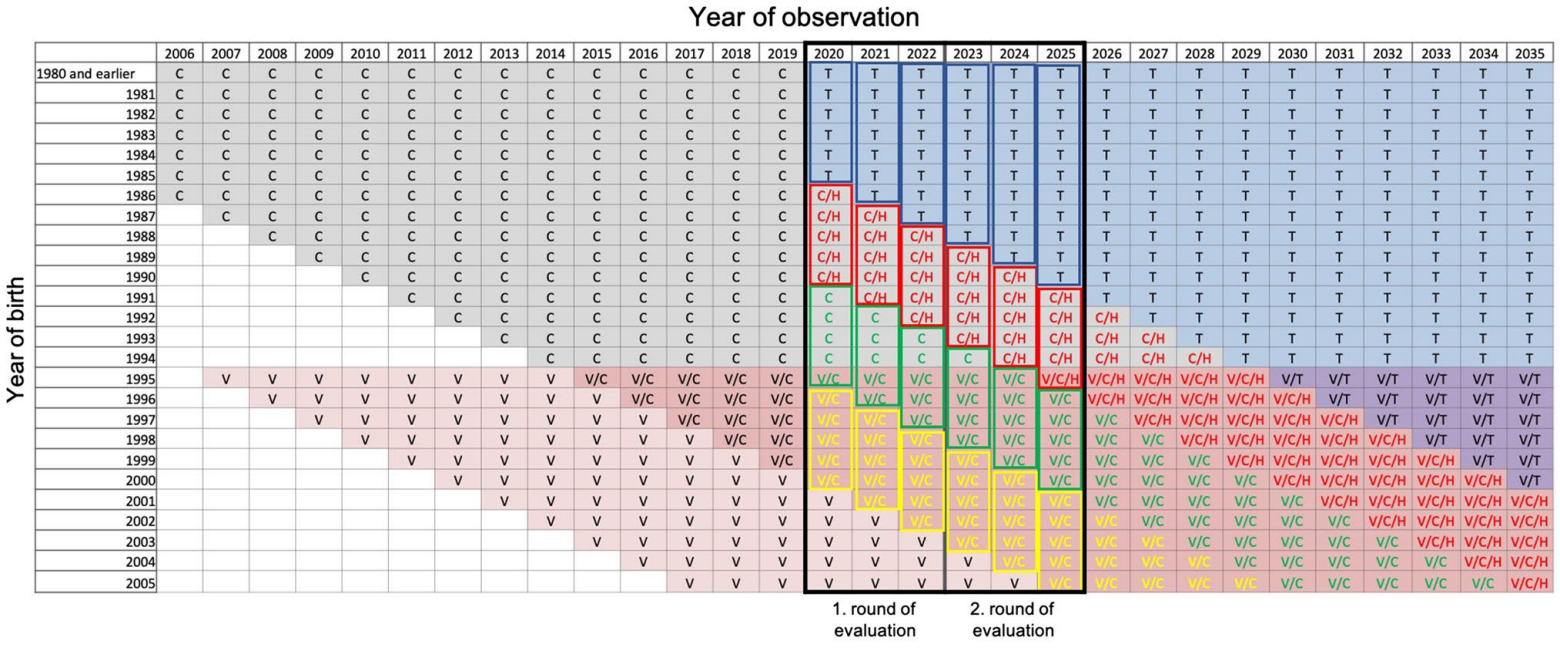
Screening plan of Germany. Availability and impact of prevention measures for individual birth cohorts in the time period 2006–2035

Yellow: 20–24 years of age; green: 25–29 years of age; red: 30–34 years of age; blue: 35 years and older

*V* vaccination, *C* cytology, *T* co-testing, *H* reflex HPV testing in case of a suspicious cytology

### Co-testing

In a proportion of women with CIN 3 present, the HPV test is positive at a time before the lesion can be detected by cytological diagnostics [6, 20, 22]. If this effect appears as requested, there will be an increase in the incidence of CIN 3 detected in the first round (so-called prevalence round). As a consequence, these CIN 3 cases should be missing in the second round. It is difficult to predict these changes with numerical accuracy in relation to the situation in Germany. However, initial publications on the impact of primary HPV screening and reviews of large studies on primary HPV testing and co-testing are available. The data range from only minor effects to multiple increases in observed CIN 3 in the first round [23–25]. According to our own experience from the German AHPV Screening Trial (GAST) [5, 20], we know that detection of CIN 3 can be doubled if all HPV-positive and cytologically negative women are clarified consequently by prompt colposcopy [5, 6, 20]. Detection rates of CIN 3 using cytologic screening alone are approximately 50% in the aforementioned studies [6, 20]. These results, along with the German guideline on the prevention of cervical cancer, were the reason for the Federal Joint Committee to adopt the oKFE-RL in its present form. Reports from Australia and the Netherlands show that the rate of additionally diagnosed CIN 3 cases raise by 30% in the first round [23, 24]. Reflecting on our experience from the German AHPV Screening Trial [5, 20], this seems to be a realistic figure. We have, therefore, adopted this for the modeling (Table 1). Since AIS and invasive adenocarcinoma of the cervix are also HPV positive in more than 90% [26, 27], we assume a similar effect for this group as well, even though it cannot be accurately estimated numerically in the present study.

The screening group of 30–34-year-old women represents a small but special group in the modeling. On the one hand, these women belong to the group with primary cytological diagnostics, and on the other hand, additional HPV diagnostics is performed within 6–12 months as a reflex in case of abnormal cytology. Due to this fact, we expect an increase of the diagnosed CIN 3 cases in this group. This increase will not occur to the same extent as in the co-testing group. Here, there is expected an increase of the cases by 15% (Table 1). In the 2nd evaluation round, the first cohort of women with access to HPV vaccination steps into this age group (Table 2). Therefore, only a proportional change in the incidence of CIN 3 was assumed here as well.

## Results of the modeling

In the time period 2014–2016, a total of 733 CIN 3 were recorded in the female population of Saarland (Table 3). Figure 1 shows the age distribution curve of these women. The curve of this distribution shows a steep increase from the age of 20 years, reaches a peak around the age of 35 years and then decreases not quite as steeply as at the beginning until the age of 50 years, finally reaching the zero line asymptomatically through a shoulder until the age of 60 years. Over the past 20 years, under the influence of cytologic screening, the age distribution of women with CIN 3 has remained largely stable.

**Table 3:** Age-specific incidence of CIN 3 (baseline) and estimated change in incidence and raw numbers (round 1 and round 2) divided into 5-year age groups (incidence per 100,000)

Age group (years)	0–14	15–19	20–24	25–29	30–34	35–39	40–44	45–49	50–54	55–59	60–64	65–69	70–74	75–79	80–84	85+	Total
Incidence of CIN 3: 2014–2016	0.0	8.4	21.1	163.8	204.3	152.5	104.3	70.1	40.4	17.0	11.8	9.1	8.4	5.2	3.2	3.3	53.9
Raw number of CIN 3: 2014–2016	0	6	17	139	167	121	88	83	54	21	13	8	7	5	2	2	733
Incidence, estimated for 2020–2022 (1. round)	0.0	5.5	13.7	140.8	234.6	198.3	135.5	91.2	52.5	22.1	15.3	11.8	7.0	6.8	4.1	2.3	62.5
Raw number, estimated for 2020–2022 (1. round)	0	4	11	120	192	157	114	108	70	27	17	10	9	7	3	3	852
Incidence, estimated for 2023–2025 (2. round)	0.0	3.6	8.9	94.4	167.5	106.8	73.0	49.1	28.3	11.9	8.2	6.4	5.9	3.6	2.2	4.2	40.0
Raw number, estimated for 2023–2025 (2. round)	0	3	7	80	137	85	62	58	38	15	9	6	5	4	1	1	511

Table 2 shows the prevention interventions offered to the female population for individual birth cohorts over the time period from 2006 to 2035. This plot illustrates which birth cohort of women receives which intervention. It shows in which age groups or screening groups effects of vaccination, cytology, and co-testing are observed and at what time.

### Changes after the first round

After the first round in 2020–2022, initial changes should already be apparent with regards to the number as well as age composition of patients with CIN 3. This applies to both the impact of vaccination and co-testing. An effect on the age group of women between 30 and 34 years of age will also be observed, even if to a smaller extent.

**Fig. 2:**
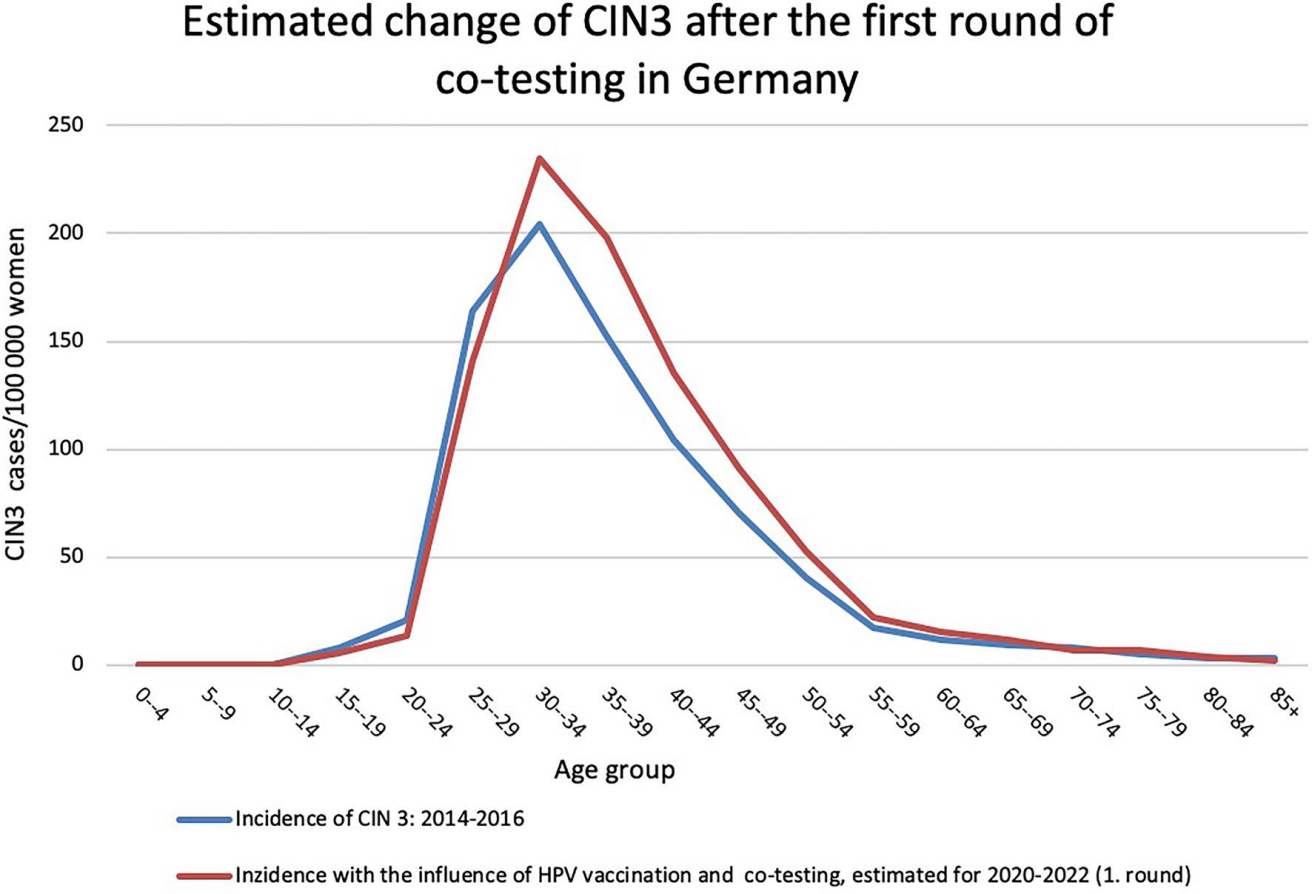
Estimated age-specific change in incidence of CIN 3 in Germany after the first screening round 2020–2022. Blue line: age-specific incidence of CIN 3 for the time period 2014–2016. Red line: estimated incidence under influence of HPV vaccination and co-testing for the time period 2020–2022. In each case as rate of CIN 3 cases per 100,000 women and year. Blue line: incidence across all age groups: 53.9/100,000 CIN 3 cases, Red line: incidence across all age groups: 62.5/100,000 CIN 3 cases

This is initially reflected in a decrease in CIN 3 cases among vaccinated women. In addition, an increase in CIN 3 should be observable in the age group of women in whom the co-test was used (Fig. 2, Table 3).

### Changes after the second round

**Fig. 3:**
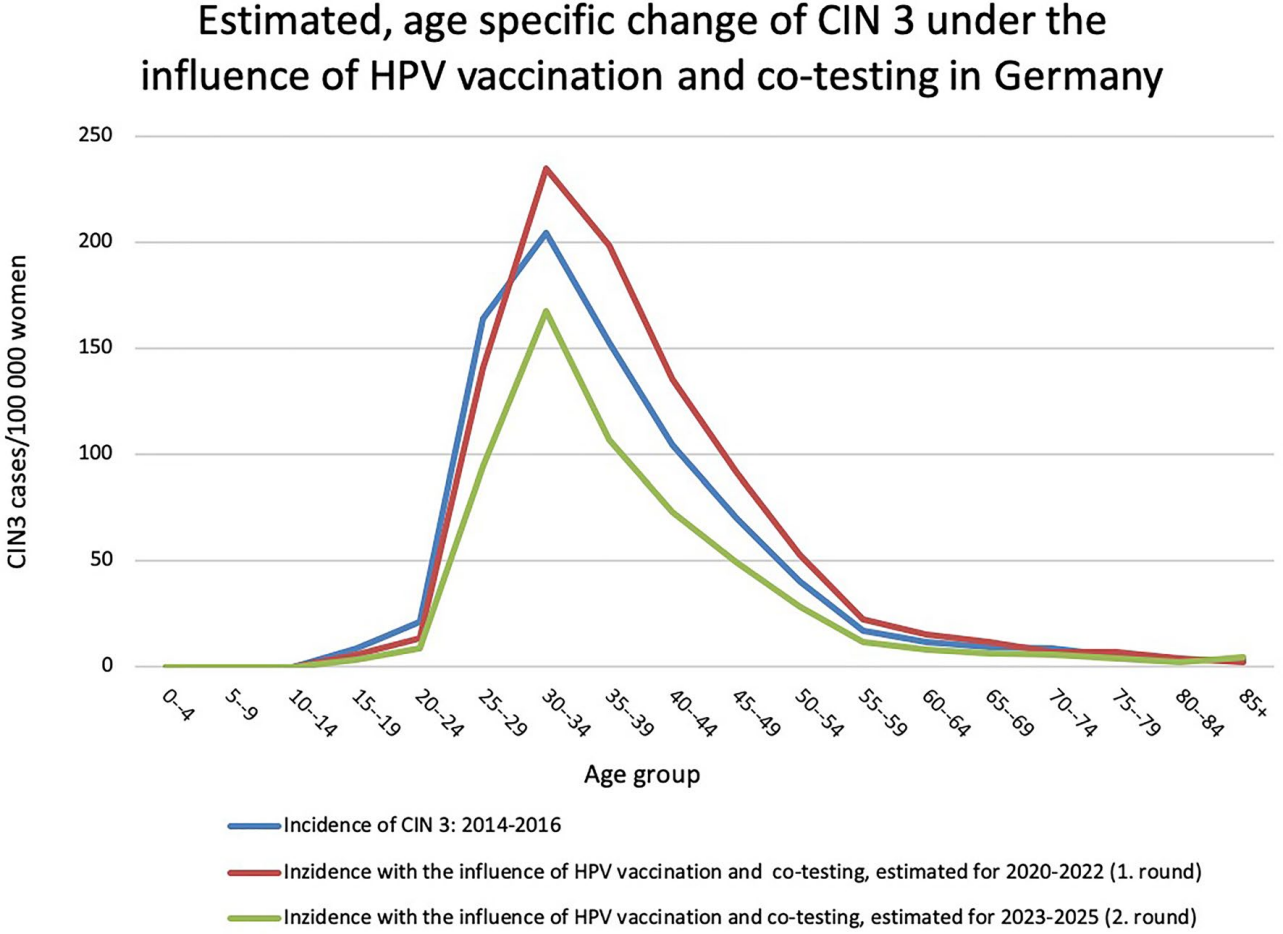
Estimated, age-specific change in the incidence of CIN 3 in Germany after two screening rounds. Blue line: age-specific incidence of CIN 3 for the time period 2014–2016. Red line: estimated incidence with influence of HPV vaccination and co-testing for the time period 2020–2022. Green line: estimated incidence with influence of HPV vaccination and co-testing for the time period 2022–2025. In each case as rates of CIN 3 cases per 100,000 women per year. Blue line: incidence across all age groups: 53.9/100,000 CIN 3 cases, Red line: incidence across all age groups: 62.5/100,000 CIN 3 cases. Green line: incidence over all age groups: 40.0/100,000 CIN 3 cases

After completing the second round, which includes the years of 2023, 2024 and 2025, the primary preventive effect of HPV vaccination will strengthen further, since the first women from the birth cohorts with access to vaccination will then already have reached the age of 30. In total, this should significantly reduce the number of CIN 3 cases in women between the ages of 21 and 29 (− 33%, see Table 1). In women between 30 and 34 years of age, the effect should be more noticeable due to the additional use of HPV testing in the screening algorithm (Table 1). In the population of women in whom co-testing was performed, two changes can be expected: on the one hand, the additional CIN 3 detected in the first round of screening are missing, and on the other hand, the addition of HPV testing continues to lead to increased detection of cytologically occult CIN 3 in round 2. Here, we expect a reduction of 30% compared to the baseline curve (Table 1). The effects in the different age groups are amplified, so that by the end of the second round, an effective reduction in CIN 3 cases has occurred compared to the time period before the new organized cervical cancer screening (Fig. 3, Table 3).

The curve impressively shows the expected reduction in CIN 3 incidence in the model. At the same time, it provides a profound insight into the population segments of particular interest with the different influences on the incidence and detection of CIN 3. It becomes visible that after a temporary increase in CIN 3 incidence in the time period 2020 to 2022 (screening round), a pronounced decrease in incidence can be expected in all age groups from the second round onwards in the modified cancer screening program. The decline in incidence of CIN 3 is particularly pronounced in the 25–29 and 35–39 age groups, as this is where the highest incidence is found.

Extrapolating the data from the Saarland Cancer Registry, the incidence of CIN 3 increases from 53.9/100,000 to 62.5/100,000 women after round 1 and will then drop to 40.0/100,000 women after round 2 (Table 3). This would be an estimated 25.8% reduction in the initial incidence of CIN 3 from 2014 to 2016 after 6 years and 2 rounds. Compared with the reference period, this corresponds to a decrease of about 5500 CIN 3 cases in the female population of Germany, if the current population size is used.

A preliminary analysis from the first 2 years of our own data from the consultation and the laboratory already support that the results we expect are in the right direction. A final evaluation will first be possible once the first round has been completed.

## Discussion

Currently, invasive cancers of the cervix represent 1.9% of all malignant neoplasms in women in Germany [9]. The conversion of the screening strategy of cervical cancer according to the oKFE-RL with a transfer to an organized system should contribute to a further reduction in the incidence of invasive cervical cancer [2]. HPV vaccination of children and young adults of both sexes has the same goal. The primary effects of both prevention strategies, the prevention or earliest possible detection of invasive cervical cancer and its precursors, can be vividly illustrated in the model we propose. Currently, the effects can still be observed separately, but they will initially overlap only partially from 2025 onward, and completely from 2030 onward. Therefore, it is reasonable to consider both prevention approaches already now.

The impact of the prevention strategy on invasive cervical cancer will only become observable in 20–30 years. Thus, in most international studies reference is made to the group of CIN 2+ [13, 22]. However, since in our case the incidence of CIN 3 is assumed to be 25,000 cases per year in Germany [28], the incidence of CIN 3 can be used as a much more valid marker for the evaluation of the presented model [29].

In the model, the reduction of the incidence of CIN 3 cases in the entire female population is vividly shown and at the same time allows a deep insight into the age groups of particular interest. The influences of HPV vaccination on the development of CIN 3 as well as the influences of the two secondary prevention mechanisms—cytology alone and co-testing—can be identified separately on the same age distribution curve. In the model-based analysis, it becomes apparent that after a temporary increase in CIN 3 incidence in the time period from 2020 to 2022, a sharp decrease in the incidence of these lesions as a precursor to invasive cervical cancer should be seen, especially in the 2nd round 2023–2025 of the new cervical cancer screening program.

The most important cornerstones of the oKFE-RL are the continued annual cytodiagnostics in women from the age of 21 and the implementation of the co-test at 3-year intervals in the female population from the age of 35 without an upper age limit. In the age group of women between 30 and 34 years of age, a transitional solution was implemented, in which the clarification of abnormal findings will include HPV testing as a reflex test. The aim of the algorithm is to establish the indication for colposcopy as early as possible, so that the sensitivity of the HPV test compared with cytodiagnostics alone, with regard to the target lesion CIN 3, comes to bear and this is, therefore, detected earlier. We assumed the influence of the co-test on the reduction of the incidence of CIN 3 to be 30% and the introduction of the HPV test in the clarification of abnormal cytological findings in the 30–34-year-old women to reduce the incidence by 15%.

### Effect of HPV vaccination

The effect of the vaccination will become visible in the age distribution curve until the 30th year of age during the observation phase until 2025. In the overall view of the influencing parameters, we estimate this effect at minus 35%. From the year 2017, instead of the bivalent or quadrivalent vaccine, the nonavalent vaccine was used. This means that from the years 2026/2027, this influence should also be taken into account.

The vaccination program in Australia has reduced the prevalence of HPV types 16, 18, 6, and 11 in the 18–24-year-old group by approximately 70% within 4 years [30]. In Scotland, the national vaccination program using bivalent HPV vaccine achieved a significant reduction in CIN 3 with a vaccination rate of approximately 90% in the cohort of women born in 1995 [31]. Overall, the vaccinated group showed an 89% reduction in CIN 3. A greater effect was observed the earlier vaccination was applied. In addition, evidence of herd protection was found. A Swedish study showed a reduction in HPV16 incidence in catchup HPV vaccinated women from 35 to 5% at 10 years of follow-up [32]. With this in mind, Hall 2018 modeled the impact of primary HPV screening as well as HPV vaccination for the Australian population [33]. The authors assume a decrease in CIN 2/3 of 40–44% and in invasive cervical cancer of 42–51% over the course of 17 years. A requirement for the strong reduction of invasive cervical cancer and its precursors is a vaccination rate of approximately 80% in both sexes. In Germany, the rate of fully vaccinated girls increased from 44.6% (2015) [34] to 52.0% (2019) [12]. In comparison with other European countries with vaccination rates of 70–90% [35], Germany is in one of the last ranks. The impact of vaccination depends on the respective vaccination rate in each federal state. In the German states, the rate of complete vaccination in the 18-year-old group in 2019 ranged from 42.0 to 70.5% [12]. As a result, the impact of HPV vaccination will be evident to different degrees in different regions of Germany. As the dropout rate in the group of 18-year-old girls is approximately 20% in Germany the average of fully vaccinated girls is only 52.0% [36]. The vaccination rate of boys is far below this. Only 2.5% of boys are vaccinated against HPV [12]. This is due the late recommendation of the Permanent Commission on Vaccination (STIKO) for the vaccination of boys, which only occurred in Germany in 2018. The second reason is that the public health insurance companies have been covering the costs of HPV vaccination for boys only since January 2019 [36]. Since then, the vaccination rate among boys has been rising. Thus, the influence of vaccinated boys will become increasingly important in the coming years. If the vaccination rates continue to rise, a similar change in CIN 3, as in Australia [30] and Scotland [31], can then be expected. According to the systematic review of Dorlet a reduction of CIN 2+ of 31–51% within a time of 5–9 years after vaccination can be expected [14].

Only the combination of the data from the respective ECRs will allow a complete picture to be drawn. Depending on the vaccination rate of both, girls and boys, the vaccination effect could have a stronger influence on the incidence of CIN 3, so that in combination with the co-test there could be a shift in the curve of CIN 3 to the right. We therefore only assumed an effect of HPV vaccination of about 35%. If the vaccination rate continues to increase significantly in the further development, a higher effect of reduction of CIN 3 in the group of young women can be expected. Based on the collection of data in the ECRs of the different states in Germany, the effect of vaccination can be calculated knowing the vaccination rate in the respective state.

### Effect of co-test

An impact on the incidence of invasive cervical cancer will not yet become apparent in the time period to 2025, as the development of invasive cervical cancer from a CIN 3 is 10–20 years or longer [3]. Appropriate results will therefore only be observable in the period from 2030 onwards. Irrespective of this, the additional use of HPV testing, predominantly in the first round, will detect not only CIN 3 but also so far occult invasive cancers, so that the incidence of invasive cervical cancers will also increase for a short time. Evidence for this can be found in several European studies [37]. Moreover, the addition of HPV testing will significantly increase the detection rate of glandular lesions (AIS and invasive adenocarcinoma) [27], especially because AIS are difficult to diagnose by cytology due to their mostly endocervical location [38].

The higher sensitivity of HPV diagnostics, however, always coupled with a stringent application of the work-up algorithm, and thus colposcopy, leads us to expect an increase in the incidence of CIN 3 in the first round (screening round). These are essentially lesions that would not have been detected with conventional cytology during the annual checkup due to their size or location [5, 6, 20, 39]. These early detected lesions are understandably “missing” in the second round [37]. However, it cannot yet be concluded that the potentially earlier detection of CIN 3 will have an impact on the number of invasive cancers arising in the future, especially in view of the long duration of carcinogenesis. It is therefore understandable that the latest Cochrane review by Koliopoulos et al. suggests that work on this issue should be initiated with longer longitudinal observation. [40].

Rijkaart showed in 2012 that the introduction of primary HPV testing leads to earlier detection of CIN 2+ and, thus, earlier treatment [41]. The number of CIN 2+ detected in the 5 year time period with 2 rounds of screening was the same in the group with primary HPV test and cytology followed by primary HPV test [41]. A recent study from England was able to show the protective value of a negative HPV test during the course [42]. Studies on the long-term course of screening with co-testing could show a persistent effect even after 10 years [5]. In newest analysis of the population of the POBASCAM study, it was shown that patients with a positive HPV test continue to have a significantly increased long-term risk of CIN 3 even after 15 years [43]. Whether the co-test in combination with the stringent implementation of the algorithm with colposcopy will be able to further reduce the incidence of CIN 3 and invasive cancer in the following rounds in Germany, can only be conclusively clarified in 10–15 years.

Chan was able to show that when the screening strategy is changed from cytology to co-testing, an fourfold increase in colposcopies is observed [25]. The indications for colposcopy were similar to those prescribed in the oKFE-RL. A significantly increased detection rate for CIN 2+ (OR 2.5) in the co-test group, as well as a significantly lower detection rate of CIN 2+ at the end of the 2nd screening round (OR 0.23) could be shown. In the German Aptima study, the use of HPV triage showed a 2.1–3.0-fold higher colposcopy rate than with cytological screening alone [20]. The detection rate of CIN 3+ was almost doubled under study conditions (OR 1.88) [20]. The same rate of increase was shown in the ATHENA study [6]. Since the clarification of colposcopies is regulated in a similarly stringent manner in Germany by the oKFE-RL, a similarly increased rate of clarification colposcopies must be expected. In 2020, Veijalainen showed a colposcopy rate of 4% in the first round in a large Finnish study of primary HPV screening, which, in turn, dropped to 2.9% in round 2 [44]. Recently, a Danish study comparing referral to colposcopy using primary HPV testing against primary cytology showed a nearly doubled detection of CIN 3+ [CIN 3+ (RR = 1.88, 95% CI 1.56–2.28) and CIN 2+ (RR = 2.19, 95% CI 1.86–2.59)] with a threefold increase in colposcopy rates [45]. They support an HPV-based screening in Denmark with a modified algorithm to reduce the rate of unnecessary colposcopies. The similarly increased colposcopy rate in Germany due to the oKEF-RL means an overtreatment of those women with uneventful colposcopies or the detection of lesions with a high rate of remission.

In the current situation concerning wars in Syria and Ukraine, the difficult question of changes in CIN 3 and invasive cancer due to migration flows is additionally raised. According to the WHO, the incidence of invasive cervical cancer is higher than that of Germany in 2/3 of European countries [46]. For example, the incidence of cervical carcinoma in Ukraine in 2020 was 14.3/100,000, which was about twice as high as in Germany (7.6/100,000). In contrast, the incidence in Syria was only 2.8/100,000 [46]. The highest incidence worldwide is on the sub-Saharan African continent. The majority of people seeking asylum in Germany come from Somalia. Here, the incidence is 25.1/100,000 women [46]. Approximately 4000 men and women come to Germany from this country every year [47]. With this amount, the number of invasive cervical cancers that change the German statistics is negligible. To what extent the migration flows change the incidence of CIN 3 and invasive cervical cancer in Germany remains to be seen.

Since in Germany every accepted emigrant is insured by the largest statutory health insurance (AOK) and, thus, has access to the cervical cancer screening program, suspicious findings are also clarified by means of colposcopy and followed up according to the algorithm. However, it will be difficult to distinguish between patients with a migration background and those with a native background. However, the effect of immigration from different incidence areas will probably be balanced out.

The incidence of CIN 3 is currently being recorded in an increasingly comprehensive and complete manner in all German ECRs and will then be available in the coming years [48]. Accordingly, by inserting these data into our model, a baseline curve can then be generated for a much larger population, which on the one hand represents the final result of the German cytology-based cervical cancer screening and at the same time can be the starting point for further evaluation of the new screening strategy.

Our proposal to use the age distribution of incidence CIN 3 to monitor the impact of the modified screening process in terms of an ecological study has several advantages. The current age distribution curve of CIN 3 has remained stable over the last 10–20 years, that means it is a curve of a cytologically well-screened population. This correlates well with the current only slight decrease in invasive cervical cancer of 2% annually [9]. Similar age distribution curves of CIN 3 are also known from Great Britain and Denmark where data for CIN 3 and invasive cervical cancer have also been systematically recorded side by side for more than 20 years [10, 49–51]. The age distribution curves of the incidence of CIN 3 and invasive cervical cancer currently also demonstrate a comparably stable situation in these countries. In Germany, this currently represents the end of pure cytodiagnostics on the one hand, but also the initial situation of the expected effects of additional HPV diagnostics and HPV vaccination.

The peak of the age distribution of CIN 3 is currently around the age of 35, thus exactly in the age group of the population where a switch between annual cytodiagnosis towards co-testing occurs in Germany. The first birth cohort with access to vaccination will reach this age in 2030, which means there is an opportunity to observe the effect of vaccination in this population over 10 years without interference. In the age group of women with annual cytological diagnostics—between the 31st and 34th year of life—the algorithm specified in the oKFE-RL additionally provides for the secondary use of an HPV test as part of the clarification of abnormal findings, in contrast to younger women. The effects of this intervention can also be easily evaluated in this age group, both individually and in conjunction with the neighboring age cohorts.

Whether the effects actually occur as modeled and expected by us can be seen when the collected data of CIN 3 are also available from other states. This is to be expected in the foreseeable future. These data will then replace our modeling. If they turn out as expected or even more pronounced, this will confirm that it was necessary to fundamentally change the cervical cancer screening process in Germany. However, if the hoped-for effects do not occur or clearly lag behind the results in other countries with a different screening model, each individual parameter of the current program will have to be reconsidered, whereby the available data, and here in particular the frequency of CIN 3 in the population, will allow a targeted correction also in subgroups. Although CIN 3 is used as a surrogate in our work, glandular lesions and invasive cancer should be considered when evaluating the results of the oKFE-RL to get a comprehensive picture of the new screening program.

## Conclusion

In summary, the age distribution curve of CIN 3 provides a platform to illustrate the effects of both vaccination and co-testing, as requested by the German National Cancer Plan, for both scientists and non-experts. It is particularly pleasing that in the next 10 years of this longitudinal presentation, the groups of primary and secondary prevention will not overlap, so that the respective effects will be visible separately. This makes it possible to take corrective action, if necessary, already during the runtime. This is even more true if the assumptions in our model are replaced by real data from the German Epidemiologic Cancer Registries in the future.

## Abbreviations

AIS: Adenocarcinoma in situ
CIN 3: Cervical intraepithelial neoplasia grade 3
ECR: Epidemiological cancer registry
HPV: Human papilloma virus
oKFE-RL: German organized cancer screening guideline
